# Is Stress Taking the Pleasure Out of Food?—A Characterization of the Food Pleasure Profiles, Appetite, and Eating Behaviors of People with Chronic Stress

**DOI:** 10.3390/foods11131980

**Published:** 2022-07-04

**Authors:** Nikoline Bach Hyldelund, Chanette Frederiksen, Derek Victor Byrne, Barbara Vad Andersen

**Affiliations:** 1 Food Quality Perception and Society Team, iSense Lab, Department of Food Science, Faculty of Technical Sciences, Aarhus University, 8000 Aarhus, Denmark; chanette.frederiksen@food.au.dk (C.F.); derekv.byrne@food.au.dk (D.V.B.); barbarav.andersen@food.au.dk (B.V.A.); 2 Sino-Danish College (SDC), University of Chinese Academy of Sciences, Beijing 101408, China

**Keywords:** food pleasure, stress, appetite, eating behavior, consumer, anhedonia

## Abstract

Psychological stressors frequently occur in modern society, and are associated with general anhedonic traits (inability to experience pleasure) and altered eating behavior. As eating behavior is largely motivated by a desire for pleasure, the Food Pleasure Scale (FPS) was introduced as a new research tool for investigating aspects of pleasure from food-related experiences. Thereby, insights on whether some aspects of pleasure are more affected by stress than others can be investigated, and can help explain why changes in eating behavior are seen when under the influence of stress. A consumer survey including *n* = 190 Danish consumers all with moderate or high levels of perceived stress was conducted to explore the perception of pleasure from food, general appetite, meal patterns, as well as specific food preferences. The study showed that the majority found pleasure in the sensory modalities of food, as well as in the ‘comforting’ aspects of food pleasure. Furthermore, the moderately stressed respondents had fewer main meals and more post-dinner snacks and night meals, as compared to before falling ill, whereas the highly stressed group showed signs of anhedonic traits and losing appetite altogether. The present study contributes to our understanding of how a common condition, such as chronic stress, can affect individual, as well as public, health.

## 1. Introduction

The incessant increase of perceived stress has been an inherent part of modern life since the condition was first described as a comprehensive health issue in 1914 by Cannon, and later by Selye in the 1930s [1,2,3]. At the same time, the technological and social evolution have made way for a modern work life that is characterized by more freedom, flexibility, and self-governance, as well as a shorter working week in terms of working hours, giving people of the Western world even more spare time than ever [1,4]. Thereby, there seems to be a paradox in the way we have organized modern work life: on one hand, to accommodate higher levels of individual freedom, and on the other hand, increasing levels of perceived stress, and health issues are registered as a result of working under modern conditions [1,4,5,6]. Hartmut Rosa describes this paradox as the result of a phenomenon called ‘social acceleration’ [1,5]. Social acceleration is an expression of how the pace of all parts of life constantly accelerates, leaving people with a feeling of insufficiency and fatigue [1,5]. In addition, Becker describes how emotions, stress, and disease have been merged in contemporary life, and so, ‘stress’ as a concept has become an important way of thinking of and describing human vulnerability difficulties in managing life experience. She describes this belief as ‘Stressism’ [6].

### 1.1. What Is ‘Stress’?

Despite the frequent use of the term ‘stress’ both in scientific and everyday speech, a standard definition of the condition is absent, and the term has been defined and redefined over decades [7,8,9,10]. A commonly used definition by Torres and Nowson defines stress as ‘the generalised, non-specific response of the body to any factor that overwhelms, or threatens to overwhelm, the body’s compensatory abilities to maintain homeostasis’ [7]. To further understand the development of the scientific notion of stress, the term can be divided into the three sub-terms: ‘stressor’, ’stress response’, and ‘allostasis’.

A ‘stressor’ can be any kind of stimuli which is perceived as stressful by the individual. Thus, it can be anything from a specific working task to a traumatic life event, such as death in the near family. This also implies that all major life events can be deemed as stressful, even the positive events of getting married, having children, and going on vacations. Therefore, many different attempts have been made at defining the stressful events of life for the purpose of being able to measure the magnitude of stress and the adaption needed [3,11,12]. Nevertheless, there is still little agreement as to what defines a stressful event [12].

The ‘stress response’ is a term in stress research which finds it origin in the work of Lazarus and Folkman [1,3,12], who focused on the appraisal of threats posed during life, and the ability to effectively ‘cope’ with these threats. From this perspective, stress occurs when an individual perceives an event as threatening or harmful, and simultaneously experiences having inadequate coping resources available [3,10,11]. If coping with the stressors is successful, the imbalance between the demands of the stressful situation and the resources needed is levelled out, and possible discontent and discomfort are reduced. Oppositely, if coping is not successful, the imbalance and discontentment will proceed and perhaps worsen, and can lead to both physiological and mental symptoms of stress, e.g., headaches, exhaustion, memory loss, or anxiety [10].

A central concept in relation to the stress response is that of ‘allostasis’, first introduced by Bruce McEwen [3,13]. Allostasis is the physiological process, by which the body responds to a stressor, adapts to that specific stressor, and thus, reaches homeostasis again. Thereby, allostasis is also the physiological and behavioral mechanisms that allow people to cope with challenges, stressors, or high demands, and once the challenge is over, shuts off and allows the body to return to a normal relaxed state. Furthermore, McEwen defines the term ‘allostatic load’ as ‘the wear and tear that results from chronic overactivity or underactivity of allostatic systems’ [13], which can have pathophysiologic consequences. Allostatic load can be relieved in many ways. Most often, people will turn to behavioral changes, such as the consumption of alcohol and tobacco, increased sleep, less physical activity, and perhaps, an unhealthy diet too [1,9,12,13].

In biological stress research, the focus is often on the sympathetic–adrenal–medullary system (SAM) and the hypothalamic–pituitary–adrenal axis (HPA), as well as the cardiovascular, metabolic, and immune systems as allostatic systems, all of which protect the body from stressors [9,11]. In addition, it is important to understand and discriminate between chronic and acute stress, as the implications of allostasis are quite diverse for the two states of stress. In the event of acute stress, allostasis systems will activate as an appropriate and beneficial reaction, allowing the body to perform fast on a high level. This primarily happens through the SAM system, by the release of stress hormones, which will increase blood pressure and heart rate, expand the heart and muscular blood vessels, as well as accelerate the metabolism of macronutrients for the release of energy [3,9,11,14]. In the case of chronic stress, a completely different effect of allostatic systems is seen. A prolonged exposure to stress has proven to cause a long and varied list of clinically defined diseases. These health effects are caused by activation of the HPA axis and alterations of health behavior and affective regulation, which can potentially result in diseases such as depression, cardiovascular diseases, diabetes, and infectious and neurodegenerative diseases [9,13,14,15].

### 1.2. Stress-Induced Eating and Altered Perception of Reward

The complex relationship between stress and eating behavior has long been acknowledged. Stress can cause irregular eating patterns, altered food behaviors and preferences, and is believed to cause alterations in the perception of pleasure from food [9,16,17]. Research has shown that being stressed can cause non-homeostatic hunger, as food consumption has proved to have calming capacities on the physiological and behavioral stress responses [9,16,17,18]. Oppositely, other studies report a reduced intake due to activation of the SAM system [8,19,20,21]. Thus, approximately 40–70% of people suffering from stress report an increase in food intake, whereas 30–60% report a reduction under stress [8,22]. Yet, the underlying psychobiological mechanisms that shape the direction of change are largely unidentified [21,23]. In the case of chronic stress, it is largely believed that the stress response is governed by an activation of the HPA axis, with a consequent rise in the secretion of glucocorticoids. Glucocorticoids directly and indirectly stimulate food intake, especially highly palatable foods, by activating secretion of the appetite hormones, insulin, leptin, and NPY [8,16]. At the same time, food intake can dampen the physiological stress response by deactivating the HPA axis [16,24]. This deactivation is very often credited to the hedonic effects of the food, as eating will activate neural substrates, such as dopamine, in a similar manner to drug abuse [16,25,26]. Dopamine functions in multiple ways; however, in the context of eating, it is first and foremost a neurotransmitter, which codes for pleasure and enhances the desire for food. Furthermore, dopamine supports the deactivation of the HPA axis [16,26]. Experienced pleasure from food may, therefore, be the main explanation for this comforting effect of food under stressful conditions. In this manner, food intake as a reaction to the physiological and behavioral stress response will concurrently stimulate the reward pathways, and thus, possibly lead to neurobiological adaptations which will promote stress-induced eating in the future too [27,28,29]. Thus, if eating is learned to be an effective coping strategy, then it is likely that highly palatable foods could become addictive, in the same manner as other highly hedonic substances (e.g., alcohol, drugs, or tobacco) [26,30,31].

Hedonia, or, in larger degree, its contradistinction, anhedonia, have been studied primarily as symptoms of mental illnesses such as depression, anxiety, and schizophrenia [26,32,33]. Anhedonia is defined as the lack of ability to perceive pleasure, and thus, the term anhedonic traits is frequently used to describe how anhedonia is expressed in the individual. Prior human studies commonly assume that relatively mild acute stressors, as well as chronic stressors, can lead to impairments in reward function, and thus, result in anhedonic symptoms [34,35]. In fact, animal studies have found that exposure to stressors induce anhedonic and depression-like behavior, as well as dysfunctions in the dopaminergic reward pathways [26,35]. In addition, it is believed that anhedonia is heterogeneous across different mental disorders, depending on which part of the reward pathway is involved [32]. For instance, in depression, anhedonia can be regarded as a transient state, whereas in schizophrenic patients, anhedonia reflects a trait-like characteristic [32]. Many different self-report scales have been constructed for measuring anhedonia, primarily in patients of mental diseases. Examples which have been widely used include the Chapman Physical and Social Anhedonia Scale, the Fawcett–Clark Pleasure Scale, and the Snaith–Hamilton Pleasure Scales [36,37,38]. Though these scales differ, what is common for all these scales is the focus on pleasure from the perspective of anhedonia, thereby only giving attention to the dysfunctional reward systems. Moreover, these scales address anhedonia from a general perspective with respect to food, which means that specific pleasure from food and food experiences is either addressed by a single question or not at all [32,39,40]. Recently, Andersen et al. (2021), at the Department of Food Science, Aarhus University and Department of Psychology, University of Chinese Academy of Sciences, developed a scale for solely evaluating pleasure from food-related experiences [40]. Thereby, it has become possible to investigate the specific relationship between pleasure perception and eating behavior in various consumer groups. Furthermore, this scale lays the foundation for a deeper understanding of why some consumers experience having issues in terms of keeping a healthy diet or making more sustainable food choices, in addition to aiding the development of strategies for alleviating these issues.

### 1.3. Purpose of this Study

Previous research by the authors of the present article has shown that acute psychosocial stress, reward, and food choice are linked via specific reward measures [41]. More specifically, it was found that ‘implicit wanting’ as a measure of unconscious craving towards specific food types increased for high-fat sweet foods when under the influence of acute psychosocial stress. In addition, the literature on the relationship between stress and eating behavior is abundant, primarily in terms of the effects of laboratory-induced stress on food choice [24,42,43]. Nonetheless, an investigation of the effect of prolonged chronic stress on perception of food-related pleasure and eating behavior has not previously been conducted. Such a study would provide beneficial insights on why people may change dietary habits during and after periods with chronic stress. With this study, we wished to investigate our hypothesis: ‘Perceived food-related pleasure can be altered when exposed to chronic stressors.’ The specific aims of this study were to explore:

- How consumers suffering from varying degrees of chronic stress perceived pleasure from food and food experiences.
- How the perception of pleasure from food, by this specific consumer group, is demonstrated in their sense of appetite, food preferences, and eating behavior.

Regarding the first aim, it was hypothesized that consumers with varying severity of chronic stress in general would have their own distinct profile of perception of pleasure from food, with explicit nuances emerging between moderately and highly stressed consumers. In addition, it was expected that these profiles would reflect pleasure preferences towards so-called ‘comfort eating’. However, whether highly stressed people in general perceive pleasure to a lower degree, or whether it is specific aspects of pleasure that may be affected, is not yet known. In relation to the second aim, it was likewise expected that the general appetite, eating behavior, and food preferences of people with chronic stress would show an affinity for highly palatable foods, an increased number of snack meals, and, in general, a larger appetite, as is described as the most common reaction to stress in the literature [16,17]. Conversely, a reduction in appetite sensations would be a possible outcome too, as studies have reported that some people reduce food intake when stressed [8,22].

## 2. Materials and Methods

### 2.1. Data Collection

A questionnaire was designed based on the items included in the original conceptual framework for the Food Pleasure Scale [40]. The original framework consisted of twenty-one items, which each represented an aspect of food pleasure. Figure S1 shows the items included in this research. First, respondents were asked to assess, in the current moment, whether or not they, in general, experience pleasure from each of the 21 items of the scale when eating food. Afterwards, they were asked to rate each item in terms of how important that item was to their sense of pleasure when eating food. They rated each item on a 100 mm Visual Analog Scale (VAS) anchored by ‘Not important at all’ and ‘Extremely important’ at the extreme ends. A complete transcript of the questions included in the Food Pleasure Scale can be seen in Table S1.

To evaluate the stress level of each of the respondents, the ten-item Perceived Stress Scale (PSS-10, Cohen et al., 1983) was utilized [44]. The PSS-10 is a global stress measure developed to assess the extent to which an individual finds their life to be unpredictable, uncontrollable, and overloaded [44,45]. The PSS-10 consists of ten questions regarding emotions experienced within the last month. The participants were asked to assess how often they had experienced the specific emotions or thoughts in question by a 5-point ordinal scale anchored by ‘Never’ and ‘Very often’. The ratings of all items were summed to create a score ranging from 0-40, with higher scores indicating a higher level of general perceived stress.

The Snaith–Hamilton Pleasure Scale (SHAPS, Snaith et al., 1995) was included to measure the general anhedonic tone of the respondents [38]. The scale was developed to be able to assess hedonic tone and its absence, anhedonia. It does so by covering four different domains of hedonic experiences: social interaction, pastimes/interests, sensory experiences, and food/drink [38]. Furthermore, the SHAPS has been translated into numerous languages, and has been validated as a precise and reliable measure of state anhedonia [38,46,47]. The SHAPS consists of 14 self-report statements, which are rated on a 4-point ordinal Likert scale: ‘1’ = ‘Definitely agree’, ‘2’ = ‘Agree’, ‘3’ = ‘Disagree’, and ‘4’ = ‘Definitely disagree’. A higher total score indicates higher levels of state anhedonia [38,46]. Examples of statements of the SHAPS are: ‘I would enjoy being with my family or friends’, and ‘I would find pleasure in my hobbies and pastimes’ [38].

In addition, the survey focused on self-reported changes in appetite and food preferences. Thus, the respondents were asked to assess their ‘general desire for food’ on a 5-point ordinal scale anchored by ‘1’ = ‘Much less desire for food now than before I was stressed’ and ‘5’ = ‘Much larger desire for food now than before I was stressed’. Furthermore, they were to choose the meals they would normally eat during a day both before being affected by chronic stress, as well as in their current state, respectively. Response variables for these questions were simple binary: ‘Yes’/’No’. The respondents were likewise asked to assess their relative preference for different food groups in terms of perceived changes in relation to their eating behavior prior to being ill with stress. An example of such a question would be ‘To which degree have You changed your intake of fruit and vegetables as compared to before being ill with stress?’. Response variables for these questions were: ‘1’ = ‘Makes up a lesser part of my diet’, ‘2’ = ‘Makes up the same part of my diet’, and ‘3’ = ‘Makes up a larger part of my diet’.

Finally, socio-demographic and health variables were evaluated. These included ‘age’, ‘gender’, ‘educational level’, ‘number of people in the residency’, ‘height’ and ‘weight’ (for calculations of BMI), ‘smoking’ and ‘alcohol consumption habits’, ‘eating disorders’, as well as their own perception of ‘personality type in relation to being an intro-/extrovert’. All of the abovementioned variables were assessed as self-reported measures. The questionnaire was online from November 2021 to February 2022, and data were collected via the Compusense^®^ Cloud software, Compusense Inc., Version 22.0.15 2022/04/11 (Guelph, ON, Canada) [48].

### 2.2. Participants and Recruitment

A total of two-hundred and nineteen Danish respondents were recruited via specific interest groups on Facebook for people suffering from chronic stress. Only people who testified to currently being affected by stress where included, as well as having a PSS-10 score ≥ 14, corresponding to a ‘moderate’ (score: 14–26) or ‘high’ (score: 27–40) perceived stress level. Furthermore, people stating to be suffering from an eating disorder were excluded, as eating disorders may well have a large impact on individual perception of food pleasure. Finally, people who characterized their stress as acute stress were excluded, as to fulfill the aims of the study. Thereby, a sample of one-hundred and ninety respondents (155 females; 35 males) with a mean age of 44.61 years (SD = 11.31) were used in this study. Characteristics of the participants can be seen in Table 1.

Ethical approval is not required for this type of study according to the National Committee on Health Research Ethics in Denmark (Section 14 (2) in the Committee Act) [49]. All respondents gave written consent to use their data prior to commencing on the questionnaire.

### 2.3. Data Analysis

Each respondent was assigned to a consumer group based on their individual PSS-10 score. Thus, *n* = 116 (61%) belonged to the ‘Moderate stress’ group, and *n* = 74 (39%) to the ‘High stress’ group. All consecutive statistical analyses were based on these two groups. Mean (±SD) or median values (IQR) were calculated for each variable for each consumer group, and results were illustrated by either bar plots based on the mean values, or by stacked bar plots showing the distributions of answers by the two groups. The normal distribution of the data was checked by the Shapiro–Wilk test, and subsequent statistical tests were chosen accordingly. Wilcoxon’s signed rank test was utilized for detecting significant differences between the two groups on numerical vales, whereas Chi^2^ tests were used for the categorical variables. For comparing changes in meal patterns before being stressed and now within each group, McNemar’s test was used. All data analyses were conducted in R Studio©, version 1.3.1093 (Boston, MA, USA) [50]. Statistical significance was set to α < 0.05 for all calculations. For 0.05 < α < 0.08, results were reported as ‘trending’ towards a significant difference.

## 3. Results

### 3.1. Sociodemographic and Lifestyle Characteristics of the Two Groups

The two groups proved to be very similar, as no significant differences could be detected in terms of gender, age, or number of people in their residencies, respectively. For educational level, differences were found, with the ‘Moderate stress’ group having significantly more people with a medium higher education (*p* < 0.001). No other differences could be seen in the sociodemographic variables.

**Figure 1:**
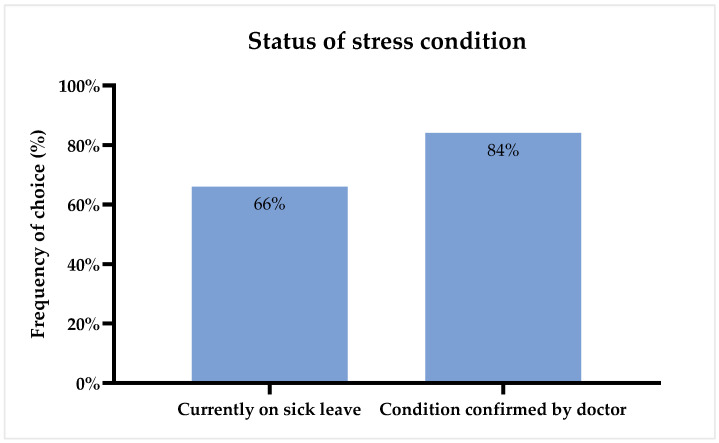
Self-reported status of condition of stress illness for all respondents (*n* = 190).

Overall, 66% of the respondents testified to currently being on sick leave due to their condition, and 84% have had their chronic stress condition confirmed by a doctor (see Figure 1).

**Figure 2:**
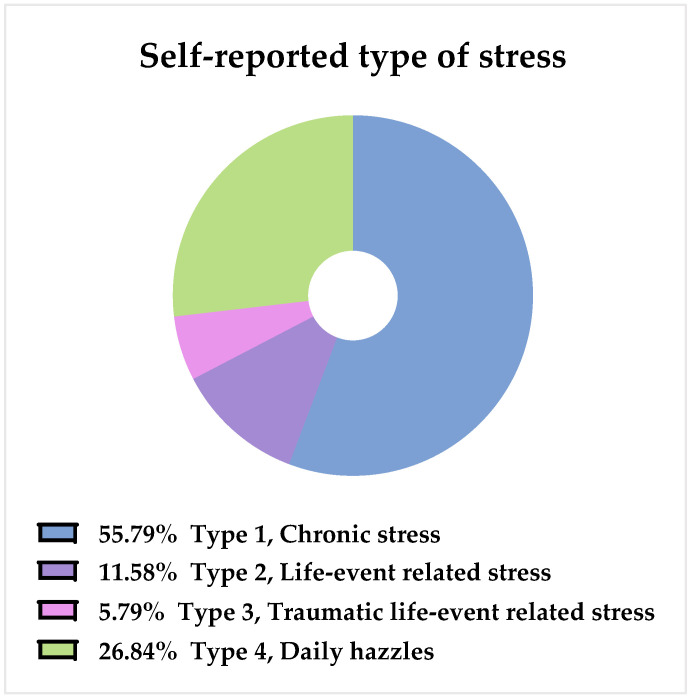
Self-reported type of stress for all respondents (*n* = 190).

Furthermore, when asked to characterize the type of stress, 56% replied ‘Type 1: Chronic stress’, whereas the three sub-types of chronic stress provided in the questionnaire were chosen as follows: 12% for the ‘Type 2: Life-event related stress’, 6% for the ‘Type 3: Traumatic life-event related stress’, and 27% for ‘Type 4: Daily hazzles’ (Figure 2). There were no differences between the two groups in these matters.

In terms of lifestyle and health factors, again, the two groups were similar, as no differences could be detected in BMI scores nor smoking and alcohol consumption habits. The ‘High stress’ group did, however, report having a significantly more introverted personality type than the ‘Moderate stress’ group (*p* = 0.011). For a complete overview of the results regarding sociodemographic and lifestyle characteristics, see Table S2.

### 3.2. Perception of Pleasure

**Figure 3:**
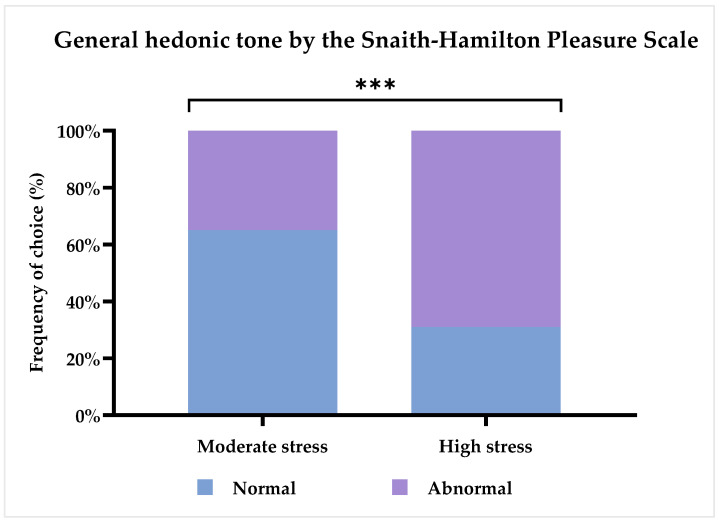
General hedonic tone by measure of the Snaith–Hamilton Pleasure Scale. Results are shown for the two groups based on the results of the PSS-10: ‘Moderate stress’ (*n* = 116) and ‘High stress’ (*n* = 74). Stars indicate level of significance of *p*-values. *** *p* < 0.001.

Over half of those surveyed were characterized as having a normal general sense of pleasure measured by the SHAPS. When taking a closer look at the SHAPS results for each group, significant differences were nonetheless found. From Figure 3 it can be seen that 65% of the ‘Moderate stress’ group had a normal hedonic score, whereas this was the case for only 31% of the respondents in the ‘High stress’ group (*p* < 0.001), thus, at the same time, bearing witness to a higher prevalence of general anhedonia in the ‘High stress’ group (*p* < 0.001).

**Figure 4:**
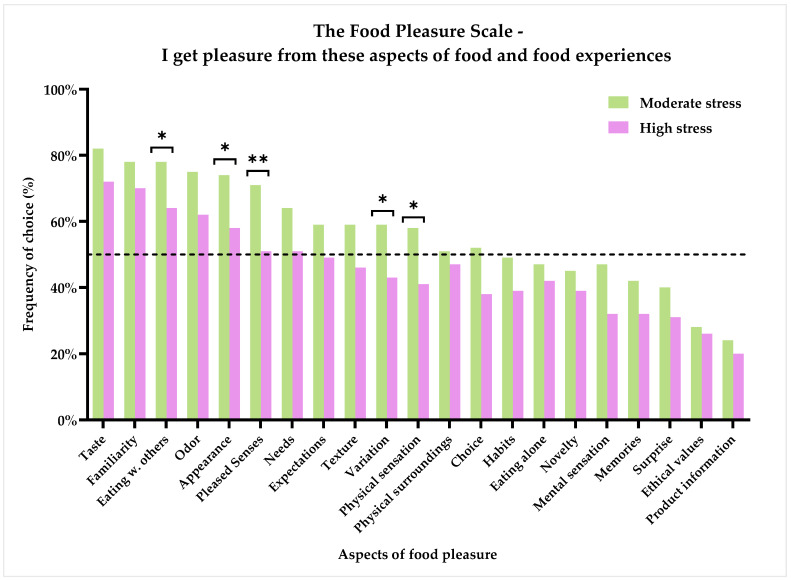
Subjective perception of pleasure from food and food experiences by the 21 different pleasure aspects of the Food Pleasure Scale. Results are shown for the two groups based on the results of the PSS-10: ‘Moderate stress’ (*n* = 116) and ‘High stress’ (*n* = 74). Stars indicate level of significance of *p*-values. *: *p* < 0.05, **: *p* < 0.01. Dotted line marks 50% frequency.

If we now turn to the results of the Food Pleasure Scale, and, more specifically, the question of which aspects of food pleasure the respondents get pleasure from, the results showed that the three most frequently chosen aspects for all respondents were ‘Taste’, ‘Familiarity’, and ‘Eating with others’, with 78%, 75%, and 72% of the respondents indicating to get pleasure from these aspects around food, respectively. Figure 4 shows the frequency rates for all aspects, and as can be seen, the ranking order by the proportion of respondents within the two groups followed a similar pattern. Oppositely, the three least chosen aspects were ‘Product information’ (*n* = 43 (23%)), ‘Ethical values’ (*n* = 51 (27%)), and ‘Surprise’ (*n* = 69 (36%)). The two groups did not agree on all aspects, and significant differences in frequency of choice could be detected for ‘Eating with others’ (*p* = 0.035), ‘Appearance’ (*p* = 0.021), ‘Pleased senses’ (*p* = 0.007), ‘Variation’ (*p* = 0.029), and ‘Physical sensations’ (*p* = 0.0021). Furthermore, a trend towards significant differences between the two groups was seen for the food pleasure aspects: ‘Odor’ (*p* = 0.060), ‘Texture’ (*p* = 0.068), ‘Choice’ (*p* = 0.061), and ‘Mental sensations’ (*p* = 0.054). In all cases, the ’Moderate stress’ group had a higher frequency of choice than the ‘High stress’ group, indicating that more respondents in the ‘Moderate stress’ group felt pleasure from these aspects around food.

**Figure 5:**
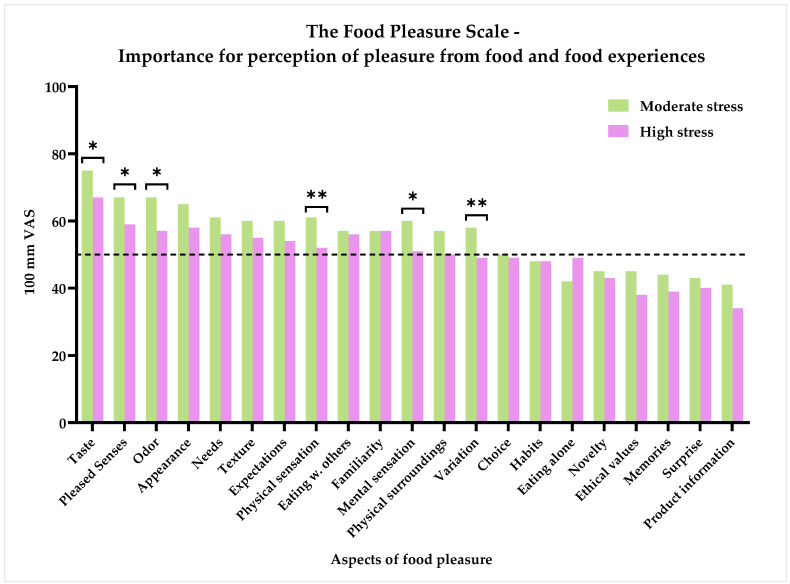
Subjective importance of pleasure from food and food experiences by the 21 different pleasure aspects of the Food Pleasure Scale rated on a 100 mm VAS scale anchored by ‘Not important at all’ and ‘Extremely important’. Results are shown for the two groups based on the results of the PSS-10: ‘Moderate stress’ (*n* = 116) and ‘High stress’ (*n* = 74). Stars indicate level of significance of *p*-values. *: *p* < 0.05, **: *p* < 0.01. Dotted line marks 50% frequency.

The respondents were asked to rate on a 100 mm VAS scale to which degree each food pleasure aspect was important for their perception of pleasure from food and food experiences. The results of this question can be seen in Figure 5. Interestingly, ‘Taste’ was rated highest by all respondents, with a mean rating of 72.59 (±20.90), followed by ‘Pleased senses’: 64.58 (±22.63), ‘Odor’: 64.01 (±22.98), and ‘Appearance’: 62.81 (±24.10). On the opposite end of the scale again, ‘Product information’, with a mean score of 38.70 (±27.65), could be seen, alongside ‘Surprise’, ‘Memories’, and ‘Ethical values’, each with mean scores of 42.02 (±24.16), 42.78 (±24.23), and 42.98 ± 27.21, respectively. Between the two groups, differences were found for ‘Taste’ (*p* = 0.021), ‘Pleased senses’ (0.035), ‘Odor’ (*p* = 0.004), ‘Physical sensations’ (*p* = 0.002), ‘Mental sensations’ (*p* = 0.015), and ‘Variation’ (*p* = 0.008). In each case, the ‘High stress’ group scored lower than the ‘Moderate stress’ group. In addition, the following aspects were trending towards significant differences: ‘Physical surroundings’ (*p* = 0.058), ‘Ethical values’ (*p* = 0.058), and ‘Eating alone’ (*p* = 0.078). Again, the ‘Moderate stress’ group scored higher than the ‘High stress’ group, with the exception of ‘Eating alone’. Interestingly, here, the ‘High stress’ group had a slightly higher mean score than the ‘Moderate stress’ group.

Together, these results indicate that when suffering from chronic stress, pleasure from food is manifested in the more basic aspects of pleasure related to the sensory perception, as well as comforting effects. Furthermore, the degree of stress seems to influence the perception of pleasure in various aspects, with higher levels of stress leading to less pleasurable aspects of food and food experiences.

### 3.3. Appetite and Meal Patterns

**Figure 6:**
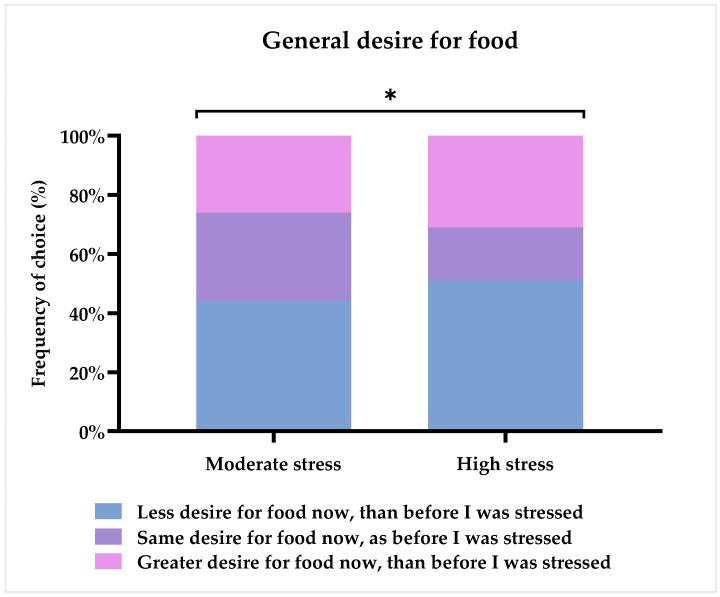
Subjective general desire for food as compared to before becoming ill with stress. Results are shown for the two groups based on the results of the PSS-10: ‘Moderate stress’ (*n* = 116) and ‘High stress’ (*n* = 74). Stars indicate level of significance of *p*-values. *: *p* < 0.05.

Respondents were asked to assess their general desire for food upon the time of conducting the study (referred to as ‘now’) as compared to before being ill with stress. Figure 6 provides the results of this question. As can be seen from the chart, almost half of all respondents have less desire for food now than before they were stressed (47%), and almost equal proportions of the remainder have either the same (25%) or a greater desire for food (28%). Taking a closer look at the answers of each of the two groups, it can be seen that the ‘High stress’ group had a smaller proportion of respondents (18%) than the ‘Moderate stress’ group (30%), who answered that they had the same desire for food. Figure 6 illustrates that there was a larger degree of diversification in answers in the ‘High stress’ group, as they had more respondents testifying to either having a greater desire for food (31%) and, especially, a lower desire for food (51%) than the ‘Moderate stress’ group. A Chi^2^-test revealed that the two groups answered differently from each other (*p* = 0.017), with the ‘Moderate stress’ group having a median value of 3 (2–4), corresponding to ‘Same desire for food now, as before I was stressed’, and the ‘High stress’ group had a median value of 2 (2–4), corresponding to ‘Less desire for food now, than before I was stressed’.

**Figure 7:**
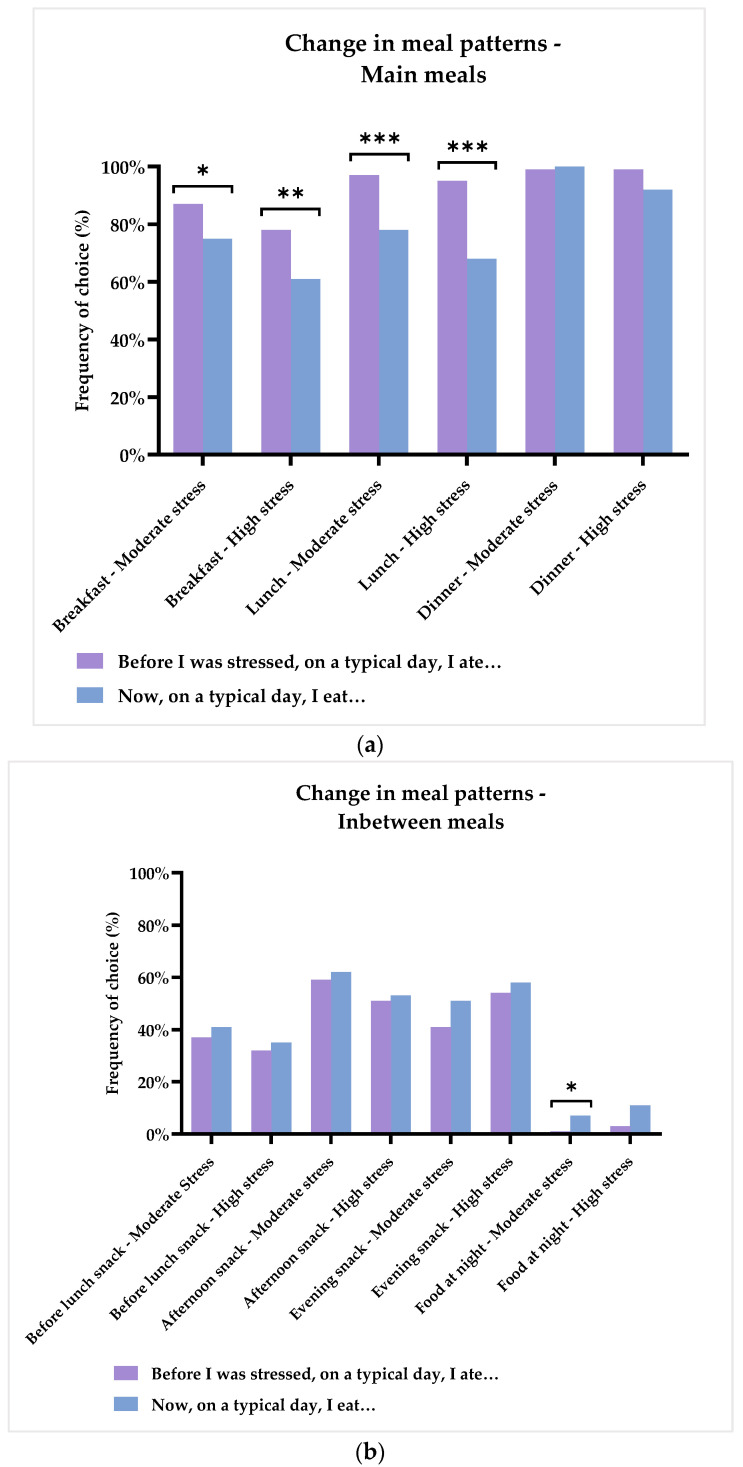
(**a**) Intake of main meals during a normal day reported by ‘Before being stressed’ and ‘Now’. Results are shown for the two groups based on the results of the PSS-10: ‘Moderate stress’ (*n* = 116) and ‘High stress’ (*n* = 74). Stars indicate level of significance of *p*-values. *: *p* < 0.001. (**b**) Intake of in-between meals during a normal day reported by ‘Before being stressed’ and ‘Now’. Results are shown for the two groups based on the results of the PSS-10: ‘Moderate stress’ (*n* = 116) and ‘High stress’ (*n* = 74). Stars indicate level of significance of *p*-values. *: *p* < 0.05, **: *p* < 0.01, ***: *p* < 0.001.

When the respondents were asked to choose which meals they had during a normal day both before being stressed and now, the majority of them reported to having at least three main meals. See Figure 7a,b for a complete overview of changes in meal patterns. Before being stressed, 99% would have dinner before being ill, with 97% testifying to having dinner now. Likewise, 96% would have lunch, whereas now, 74% eat lunch, which was a significant decrease (*p* < 0.001). This general decrease is found in both groups, as the ‘Moderate stress’ group decrease their lunch intake by 19% (*p* < 0.001), and the ‘High stress’ group by 27% (*p* < 0.001). Furthermore, 84% of all respondents would have breakfast, whereas now, only 69% eat breakfast. This is a significant decrease of 15% (*p* < 0.001), and the decrease can be seen in both of the groups (‘Moderate stress’: *p* = 0.014, ‘High stress’: *p* = 0.006), but with a larger fall in the ‘High stress’ group. In terms of snack meals, just over half of all respondents had a pre-dinner snack both before (56%) and now (58%). Similarly, approximately a third of all respondents would have a pre-lunch snack both before (35%) and now (38%). For the post-dinner snack, an increase is seen from 46% before being stressed to 54%. On a general level, no differences could be detected for any of the snack meals. The ‘Moderate stress’ group did, however, show a tendency towards an increase in the post-dinner snack by 10% (*p* = 0.074). Finally, in general, an increase was detected for having a meal during the night, with 2% and 8% having such a meal before and now, respectively (*p* = 0.004). This increase should mainly be accredited to the ‘Moderate stress’ group, as 6% increased their night meals (*p* = 0.046), whereas for the ‘High stress’ group, it was trending towards a significant increase (*p* = 0.077).

In summary, these results suggest that the general desire for food was indeed negatively influenced by chronic stress. More specifically, changes could be detected in meal patterns, with a decrease in the intake of breakfast and lunch, as well as a tendency for fewer eating dinner meals in the ‘High stress’ group. In the ‘Moderate stress’ group, significantly more ate night meals, and showed a tendency towards more post-dinner snack meals.

### 3.4. Specific Food Preferences

**Figure 8:**
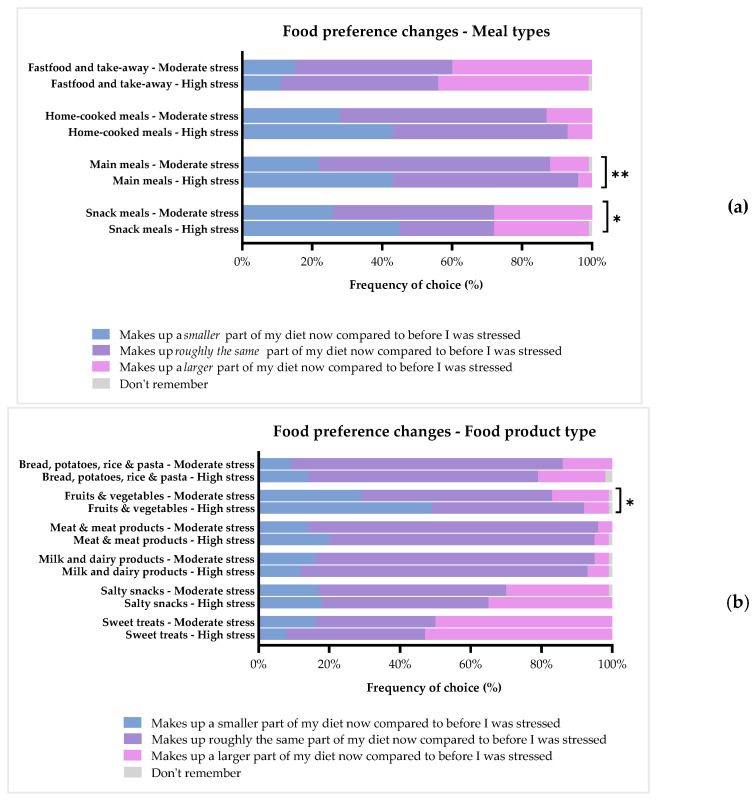

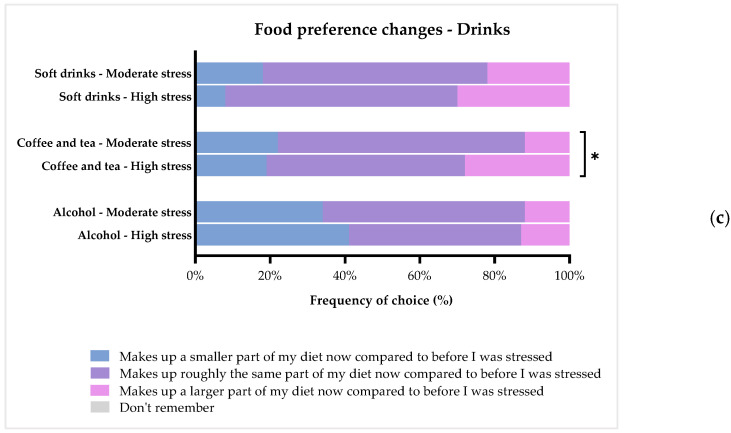
(**a**) Meal types. Diet composition reported by current state as compared to before being ill with stress. Results are shown for the two groups based on the results of the PSS-10: ‘Moderate stress’ (*n* = 116) and ‘High stress’ (*n* = 74). Stars indicate level of significance of *p*-values. *: *p* < 0.001. (**b**) Food product types. Diet composition reported by current state as compared to before being ill with stress. Results are shown for the two groups based on the results of the PSS-10: ‘Moderate stress’ (*n* = 116) and ‘High stress’ (*n* = 74). Stars indicate level of significance of *p*-values. *: *p* < 0.001. (**c**) Drinks. Diet composition reported by current state as compared to before being ill with stress. Results are shown for the two groups based on the results of the PSS-10: ‘Moderate stress’ (*n* = 116) and ‘High stress’ (*n* = 74). Stars indicate level of significance of *p*-values. *: *p* < 0.05, **: *p* < 0.01.

To get further insights into more specific dietary changes as an effect of being stressed, the respondents were asked to assess to which degree being ill with stress had changed their intake of specific food groups. Figure 8a–c illustrates changes in food preferences for the two sub-groups: moderate and high stress.

#### 3.4.1. Meal Types

Figure 8a shows the results of food preference changes regarding different meal types. More than half of all respondents reported they had the same consumption level of main meals (61%) now as compared to before being ill, whereas 31% said they had fewer main meals, and 8% had more. The two groups did not show the same consumption pattern here, as 66% of the ‘Moderate stress’ group reported having the same level, 22% had less, and 11% had more main meals. Oppositely, only 53% of the ‘High stress’ group reported to have the same level, with 43% having fewer main meals, and 4% having more. These differences proved to be significant by a Chi^2^-test (*p* = 0.006), and the results mimic those described above in Section 3.3.

For snack meals, the overall results were that 38% had the same intake level, 33% had less snack meals, and 28% had more. Again, the two groups proved to have different consumption levels for this meal category (*p* = 0.012), with 45% of the ‘High stress’ group reporting to have less snack meals as compared to the ‘Moderate stress’ group, where only 26% had less. Oppositely, 46% of the ‘Moderate stress’ group had the same level, whereas only 27% of the ‘High stress’ group reported the same. Similar proportions of the two groups reported they had more snack meals, with 28% and 27%, respectively.

Almost half of the respondents (45%) reported that they had the same intake of fast-food and takeaway now as before being ill, with 41% testifying to have more, and 14% to have less. The two groups did not differ in terms of this meal type category. Among the respondents, 55% had the same level of home-cooked meals as before, with 34% saying they have less home-cooked meals, and 11% having more. A tendency towards a difference in consumption patterns for this meal type could, however, be detected (*p* = 0.063). The difference was found in that 43% of the ‘High stress’ group reported to have fewer home-cooked meals, whereas 50% had the same, and 7% had more. In total, 28% of the ‘Moderate stress’ group reported to have fewer home-cooked meals, whereas, in turn, 59% and 13% of this group had either the same or more.

#### 3.4.2. Food Product Types

Overall, the respondents reported that stress did not change their intake of meat and meat products, milk and dairy products, nor bread, potatoes, pasta, and rice (Figure 8b). All of these food product types were reported at the same intake level for approximately 75% of the respondents. No differences in intake of these categories could be seen between the two groups either. In terms of fruit and vegetable consumption, only 50% of the respondents answered they had the same intake as before being ill with stress, and 37% reported to eat less fruit and vegetables now. The ‘High stress’ group especially decreased their intake of this food category, as 49% reported to eat less now compared to before. This was a significant decrease compared to the ‘Moderate stress’ group, with 29% reporting to eat less fruit and vegetables (*p* = 0.014). For salty snack products, 51% of all respondents said they eat the same amount, whereas 32% eat more, and 17% eat less as compared to before being ill. Sweet treats, on the other hand, were reported as being consumed more by 51%, at the same level by 36%, and to a lesser extent by 13% of all respondents. There were no differences in salty snack and sweet treat consumption between the two groups.

#### 3.4.3. Drinks

Figure 8c shows the consumption patterns for three different categories of drinks. The consumption of soft drinks, such as juice, soda, and lemonade, did not change for 61% of the respondents, whereas 25% reported to drink more of these, and 14% drank less. For alcoholic beverages, 51% of all respondents reported they had the same consumption level, 36% had less, and 13% had a higher level as compared to before being ill. The two groups had similar consumption patterns for both the soft drinks and alcoholic beverages, and as such, no differences could be detected. Overall, 61% had the same intake of coffee and tea, with 21% and 18% having less or more, respectively. The two groups differed in this category, as within the ‘Moderate stress’ group, 66% reported to have the same, 22% to drink less, and 12% drank more. On the other hand, among the ‘High stress’ group, 53% had the same intake, whereas 19% had less, and 28% drank more.

## 4. Discussion

The relationship between stress and eating behavior evidently needs more attention, so as to truly understand why healthy eating behaviors, in general, are sacrificed when people fall ill with stress [17,20,41]. When one considers the recent reported health state of Danes and other Western societies with decreasing mental health and increasingly perceived stress levels, as well as increasing obesity rates [51,52], investigations of this issue seems more imperative than ever. With the present study, a characterization of people suffering from chronic stress in relation to the perception of pleasure from food, appetite, meal patterns, and food preferences has been investigated by the use of a self-report questionnaire administered to only people currently suffering from the condition, as well as with different levels of severity (moderate vs. high perceived stress levels). Thus, this study has yielded insights into the perception of food pleasure and eating behavior of a specific consumer group during a time of their lives where anhedonic traits are presumed to be prominent. Overall, the present study found that people with a moderate or high perceived stress level do experience alterations in the perception of pleasure from food, general desire for food, meal patterns, and intake of specific food product categories, as compared to before being subjected to the condition. Furthermore, these alterations proved to be influenced by the severity of the stress condition, being moderately versus highly stressed.

### 4.1. Altered Hedonic Tone and Food-Related Pleasure as a Consequence of Stress

The results of the SHAPS showed that there were a significantly larger proportion of people with an abnormal general hedonic tone in the ‘High stress’ group compared to the ‘Moderate stress’ group. This was an interesting result, as it indicates a negative correlation between stress severity and the ability to perceive pleasure. Previous studies using the SHAPS have found a negative relation between various mental diseases, including depression, substance dependency, and schizophrenia, and hedonic tone [46,53,54]. Thus, this result implies that hedonic tone can be influenced by other mental illnesses, such as chronic stress. However, it is still unclear which aspects of pleasure, and, in particular, which aspects of food pleasure are affected by stress, and whether this knowledge would be applicable in understanding the changes observed in people’s eating behavior.

An initial objective of the study was to identify the food pleasure profiles of people suffering from different degrees of chronic stress: moderately and highly stressed. By applying the Food Pleasure Scale, insights into how various aspects of food pleasure are perceived by this specific consumer group have been attained. Interestingly, in the two groups, the majority showed to mainly experience pleasure from the same elements of food. Namely, ‘Taste’, ‘Familiarity’, ‘Eating with others’, ‘Odor’, ‘Appearance’, ‘Pleased senses’, and ‘Needs’ were all chosen by more than 50% in each group, and the ranking order of importance of these aspects also followed the same pattern, with ‘Taste’ ranked as the most important aspect around food for perceiving pleasure. For the ‘Moderate stress’ group, the aspects of ‘Expectations’, ‘Texture’, ‘Variation’, ‘Physical sensations’, ‘Choice’, and ‘Physical surroundings’ were likewise chosen to provide pleasure by more than 50% of the group. Thus, this group displayed a larger range of food-related aspects which they perceived pleasure from compared to the ‘High stress’ group. These results align with the results of importance of each aspect to perceived pleasure from food, as the ‘Moderate stress’ group likewise had more aspects they rated above the center of the VAS, and they had a higher proportion of respondents with a normal general hedonic tone.

It is interesting to note that the two groups showed a similar pattern regarding the seven most chosen aspects providing pleasure from food, and that the main theme of many of these aspects is that they belong to the sensory modality of food pleasure perception. This result support previous findings on drivers of food-related pleasure and satisfaction from food [55,56,57,58,59]. Furthermore, these results emulate those found in previous studies using the Food Pleasure Scale [60,61]. Here, the sensory aspects were likewise shown as the main drivers of food pleasure, yet among normal healthy consumer segments. The reason for this is probably the fact that everyday talk about food often focuses on the flavor/taste of the food [55,62]. Therefore, other aspects automatically become secondary for many people. Moreover, these results could also be an indication of the perception of food pleasure being linked to more stable personality-dependent traits. Thus, it could be speculated that for a shift in food pleasure preferences to happen, more intrusive life conditions are required. Further research in this hypothesis is needed to fully understand the underlying factors which are at play here.

Among the top-rated aspects, ‘Familiarity’, ‘Eating with others’, and ‘Needs’ were also found. These results suggest that for people with chronic stress, an element of consolation (of one’s emotions) by eating food that is known and ‘safe’, perhaps in the company of others, is what drives pleasure from food, and this is where this consumer group differentiates from the ‘normal’ healthy consumer. At the same time, these aspects also reflect basic human needs of feeling safety and security, as described by Maslow in his famous ‘Hierarchy of Needs’ [63]. Furthermore, fulfilling one’s needs from food could be an expression of having a craving that needs to be satisfied, or it could simply refer to a basic need of re-energizing. Thereby, the term ‘comfort eating’, which has traditionally been strongly associated with stress-induced eating [8,24,30], may apply to this sample too. The more cognitively advanced aspects of food pleasure, such as ‘Product information’, ‘Ethical values’, and ‘Surprise’, were all among the least chosen aspects, as well as the least important, for both groups, thereby suggesting that pleasure from food for this consumer group was to be effortless and non-demanding. These specific aspects of food pleasure have, in recent literature, been characterized as secondary (or tertiary) [60,61] for food liking and pleasure. Thus, it is likely this consumer group have rated these as the least important for the same reasons.

Even though there seems to be a pattern in terms of which aspects were chosen to provide pleasure to the most and least people in the sample, discrepancies could also be identified when comparing ratings of importance of each aspect for food pleasure. For the ‘Moderate stress’ group, ‘Physical sensations’, ‘Texture’, ‘Mental sensations’, and ‘Choice’ were all rated with a score above 50 on the 100 mm VAS scale, thereby indicating these aspects were important to them, yet less than 50% of the group said these aspects currently gave them pleasure. The ‘High stress’ group likewise rated ‘Texture’, ‘Physical sensations’, ‘Mental sensations’, and ‘Physical surroundings’ above 50, indicating a higher than neutral importance of these aspects to food-related pleasure, whereas less than half of this group testified to currently get pleasure from these aspects. It can be hypothesized that the ability to perceive pleasure from these aspects, of which some can be characterized as interoceptive sensations, becomes impaired, or at least less important, when affected by stress. Further work is required to establish the viability of this hypothesis; however, that would require a more substantial survey on this consumer group. As stated in the results, differences were evident between the two groups in terms of how often an aspect was chosen as to provide pleasure, as well as the importance of each aspect for the perception of pleasure. The ‘Moderate stress’ group had significantly more respondents choosing ‘Appearance’ (*p* = 0.021) and ‘Pleased senses’ (*p* = 0.007), and they rated ‘Taste’ (*p* = 0.021), ‘Pleased senses’ (*p* = 0.035), ‘Odor’ (*p* = 0.004), ‘Mental sensation’ (*p* = 0.015), ‘Physical sensations’ (*p* = 0.002), and ‘Variation’ (*p* = 0.008) higher in terms of importance. Taken together, the ‘High stress’ group seems to be showing anhedonic traits that go beyond the focus of attaining food pleasure from just sensory and comforting aspects. The results in this chapter indicated that higher perceived stress levels impaired the perception of pleasure from food. The next chapter, therefore, moves on to discuss how this is manifested in eating behaviors and food choices.

### 4.2. The Effect of Chronic Stress on Appetite, Meal Patterns, and Food Preferences

In general, the study showed that chronic stress influence meal patterns and specific food preferences, especially in relation to main and snack meals. Increases in sweet and salty snacks and caffeinated drinks, as well as decreases in the intake of fruit and vegetables, were found. More specifically, when investigating the effects of stress severity, it was found that the ‘High stress’ group had a lower desire for food than the ‘Moderate stress’ group. This result was reflected in the changes of meal patterns, as the ‘High stress’ group had a larger decrease in intake of both breakfast and lunch, as well as snack meals and, particularly, fruits and vegetables. Like the ‘High stress’ group, the ‘Moderate stress’ group also showed a decrease of breakfast and lunch meals, but to a lesser degree. Additionally, more respondents suffering from ‘Moderate stress’ compared to ‘High stress’ demonstrated an increase in night meals and a tendency towards more post-dinner snacks. These results exemplify, in a discrete way, what the scientific literature has stated for years, namely, that when experiencing chronic stress, approximately 40–70% increase their food intake, whereas 30–60% reduce their intake [8,16,17]. Yet, the psychobiological mechanisms which shape the course of this change are largely unknown (which is why it has not been fully explored), as well as which factors account for these individual differences. Furthermore, several papers have reported that chronic stress is linked to a change in diet towards fewer main meals and higher intake of, especially, highly-palatable snack foods, mainly due to a continued activation of the HPA axis [16,64]. In line with these studies and a previous study on acute stress reporting an increase in the unconscious craving for high-fat sweet snack foods [41], the majority of respondents reported to have increased their intake of sweet snacks after being ill with stress, and a large group likewise reported to have increased their intake of salty snacks.

The food pleasure profile of this sample can be used to expand the understanding of this change in diet. As the most important aspect to food pleasure came from the sensory modalities of the food eaten, it makes sense that foods that have a strong sensory output are chosen more often. Snack foods are generally known to be highly satisfactory both in terms of sensory profile, as well in terms of well-being sensations, giving instant relief from the stress condition [16,42,64]. Conversely, main meals are often chosen and composed upon reasoning around nutrition; the social context, which requires product information; and, potentially, considerations around ethical values. These aspects around a meal require effort to fulfill. Thus, it seems logical that these meals are eaten less, and that these aspects provide pleasure to few people when mental resources (e.g., requirements to cognitive efforts) are low, such as those in the current stress conditions. It can be hypothesized that the same goes for fruit and vegetable intake. Again, this food group represents nutrition and health for many people, and making a healthy choice can seem insuperable under the influence of stress, especially if the condition is at a level which involves the activation of the HPA axis and secretion of glucocorticoids.

One would assume the ‘High stress’ group would follow the same change in eating pattern of reducing main meals while also increasing intake of snack foods, perhaps with an even more pronounced dietary change. However, as seen from the results, this group only follows the pattern in terms of main meals, and thus, not in the intake of snack meals. The severity of their condition can explain these results, as more severe stress does, to a larger degree, impose anhedonic traits, as it is often seen in people with depression and other mental disorders [26,32,65]. Anhedonic traits were also reflected in the results from the FPS, as the ‘High stress’ group had a narrower range of aspects providing pleasure, and, in general, rated the aspects lower in terms of importance for pleasure. A loss of appetite as a mere symptom of anhedonia, and thus, may be the cause for their dietary pattern. Therefore, it seems that the dietary changes of the ‘High stress’ group are not as much of an expression of the HPA axis being activated for a prolonged period of time, but a consequence of a condition that is leaving deeper marks in a person’s mental health (ability to perceive pleasure). Further research into the food pleasure profiles and eating behavior patterns of people known to have anhedonic traits is highly recommended to be able to fully understand the impact of mental health on dietary habits and food choice.

Interestingly, the ‘High stress’ group increased their intake of caffeinated drinks. A positive correlation between occupational stress and coffee consumption has been established years ago [66,67,68], yet why this relationship exists is not quite clear. As the highly stressed group displayed a larger degree of anhedonia, it seems reasonable that they, to some degree, experience an aversion towards food. A simple thing such as a cup of tea or coffee may again be an easier choice for relief of hunger and low energy. Moreover, having a cup of coffee or tea may seem more manageable than cooking up a complete meal. A ten-year longitudinal study including a total of 50,739 US women showed that depression risk decreases with increased caffeinated coffee consumption [66]. Thus, the increase in coffee consumption of the highly stressed group could, in fact, be a positive finding, especially as caffeine has been shown to enhance dopaminergic activity in animal studies, as well as to cause increased well-being, energy, and alertness in human behavioral studies [69,70]. Caffeine may thus be used by the highly stressed group as a way of compensating for anhedonic or depression-like symptoms.

### 4.3. Implications

The present study has yielded insights into the appetite, eating behaviors, and, not least, perception of food pleasure of a consumer group who have not been studied in any great detail before. Links between stress and eating behavior, previously described in the literature, have been confirmed [8,16,17,20], whereas new insights regarding the more severe condition of chronic stress have emerged. In particular, the distinction between people of moderate and high chronic stress levels proved to be fruitful in terms of a more nuanced exploration of the condition and its impact on the perception of pleasure and eating behavior. Thereby, valuable insights have been found for future research in the subjects of mental illness, (food) anhedonia, and consumer behaviors.

Stress has proven to be one of modern time’s biggest health issues, with ever-increasing rates of mainly the chronic version of the condition [51,52,71]. The latest National Danish Health Report was published in 2022, and a major issue highlighted was the mental health situation, with increasing rates of depression, anxiety, and stress, especially among young people [51]. The condition appears to be grounded in the very structure of modern society, and thus, complete prevention seems to require multiple and highly comprehensive means [1,5,10]. Nonetheless, there is abundant room for further progress in research focusing on the prevention of the repercussions of the condition, as well as in public health interventions. This study offers valuable insights for use in the public health sector in the work of guiding consumers in relation to dietary habits when falling ill with chronic stress. Having a deeper knowledge of what drives the individual perception of pleasure from food can be used as an active tool in the treatment/management of stress, as an increased general wellbeing may relieve some of the symptoms of the condition. Likewise, this knowledge can be used in the prevention of known long-term health effects of living with chronic stress, such as unhealthy eating, possibly leading to severe health complications. Thereby, possible malnutrition or weight-gain can be prevented, ultimately securing the health and strength of the consumers to overcome the condition. On a practical level, health professionals can use the results of the current study as a guide for counselling on diet and eating behavior based upon the severity of the condition, as well as the individual food pleasure profile of the patient. Thereby, the alleviation of anhedonic symptoms can increase physical health and, potentially, also mental health.

### 4.4. Strengths and Limitations of the Study

To the authors’ knowledge, no previous studies have investigated the perception of pleasure, appetite, and eating behavior in a human study with a sample of people suffering from chronic stress. Furthermore, the stratification of a sample by PSS-10 scores has not been seen before, which allowed an understanding of the effects of chronic stress differentiated by severity. This study, therefore, offers unique insights into the everyday food-related consequences of dealing with chronic stress. Another strength of the study was the fact that the sample consisted of consumers currently under the influence of chronic stress when the survey questionnaire was completed. Thus, a nuanced snapshot at a difficult time of life of this specific consumer group has been achieved, and the study has not relied on artificially inducing stress, as other studies have previously done, to measure the effect of stress on eating behavior [20,22,42,64].

This study was an online consumer questionnaire based on subjective self-report responses. This methodological approach offers both possibilities, as well as limitations. One important limitation is that results are dependent on retrospection, especially for the questions regarding appetite and eating behavior before falling ill. Likewise, there is the well-known self-report bias of respondents tweaking answers for a better fit of their own self-perception [72]. Thus, as the respondents report having a less healthier eating behavior now as opposed to before falling ill, the possibility that the respondents actually have an even less healthy diet now than that reported in the questionnaire should not be excluded. Nevertheless, the authors were under the impression that the consumers choosing to participate in the study were interested in contributing to the research in the field of stress, so as to benefit future public health efforts. Furthermore, designing a survey questionnaire for this specific consumer group demands a high level of precaution in terms of not exhausting nor overwhelming the respondents. Thereby, the length and complexity of the survey were meticulously cared for. This ensured a concise and simple questionnaire, yet, at the same time, reduced the possibility for harvesting interesting insights on a consumer group who can be hard to reach in the first place. Further studies on, e.g., the perception of interoceptive sensations or the psycho-behavioral effects of stress by implicit measures of food reward of this specific consumer group, would be very highly beneficial for truly understanding the effect chronic stress has on individual health and well-being. Finally, it must be noted that the current study population consisted of 82% females. The study does not address gender differences, as the focus chosen was on the severity of the condition. A study with a larger sample size including more males is thus recommended to check the generalizability of the results. Furthermore, it is advisable to perform additional studies including ‘non-ill’ participants, to further investigate whether the food pleasure profiles discovered in this study truly were distinct to this consumer group.

## 5. Conclusions

This study aimed to explore the food-related pleasure, appetite, and eating behaviors of consumers suffering from different degrees of a chronic stress condition. These aspects were addressed in relation to the sample’s own perception of prior experiences, thus emphasizing the subjective consciously perceived changes as a consequence of stress. By doing so, the ‘stress-eating behavior’ relationship has been investigated from a diversified approach to chronic stress. Overall, the study showed that both groups found pleasure in the sensory modalities of food, as well as in aspects of food pleasure, which can be ascribed to a comforting effect, i.e., a sense of familiarity or having basic needs of safety and security met. Oppositely, more advanced aspects of pleasure, such as ethical value, product information, and surprises, did not prove to yield pleasure, nor had importance to the respondents. More specifically, the highly stressed respondents, in general, showed a narrower range of aspects from which they attained food pleasure, and, in general, rated all food pleasure aspects as less important to them than the moderately stressed. The ‘high stress’ group likewise proved to have less general desire for food, and had a larger proportion of respondents with an abnormal SHAPS score than the moderately stressed group.

The most obvious finding to emerge from this study is that the moderately stressed respondents exhibited a change in eating behavior and food preferences towards less main meals and more post-dinner snacks and night meals, whereas the highly stressed group showed signs of anhedonic traits and losing appetite altogether. Taken together, these results suggest that different levels of perceived stress not only affect the perception of pleasure from food, appetite, eating behavior, and food preferences, but can leave deeper marks on a person’s mental health, with the possibility of negatively affecting wellbeing and physical health as a consequence of anhedonia.

The present study contributes to our understanding of how a common condition, such as chronic stress, can affect individual, as well as public, health. Insights from this study could be the key to better understanding why a loss of control of diet is experienced by many people in post-modern society. Moreover, the results of this study could serve as a guide for public health professionals in the work of guiding patients on diet and eating behavior based upon the severity of their condition, as well as their individual food pleasure profile. Further research in the food pleasure profiles and eating behaviors of people known to have anhedonic traits are needed to fully understand the impact of mental health on dietary habits and food choice, and, ultimately, health and wellbeing too.

## Figures and Tables

**Table 1:** Characteristics of participants.

Characteristics	Title
*n*_total_	190
Males/females (%)	35 (18%)/155 (82%)
Age (years) *	44.61 ± 11.31 (23–67)
Educational level	
Primary school (%)	5 (3%)
High school (%)	12 (6%)
Vocational education (%)	43 (22%)
Short higher Education (%)	28 (14%)
Medium higher Education (%)	68 (36%)
Long higher Education (%)	33 (17%)
PhD	1 (1%)
BMI (kg/m^2^) ^1,^*	28.00 ± 6.40 (18–53)
Perceived Stress Scale (PSS-10) ^2^	
Moderate stress (%)	116 (61%)
High stress (%)	74 (39%)

* Mean ± standard deviation (range), ^1^ BMI: Body Mass Index, ^2^ PSS-10: Perceived Stress Scale. Moderate stress level corresponds to a score of 14–26, high stress level to a score of 27–40.

## Data Availability

The datasets generated for this study are available upon request to the corresponding author.

## Supplementary Materials

The following supporting information can be downloaded at: https://www.mdpi.com/article/10.3390/foods11131980/s1, Figure S1: Schematic conceptualization of the key dimensions, items, and behavioral elements involved in the individual food-related pleasure response, allowing a holistic study of quantitative (level of pleasure) and qualitative (drivers of pleasure) aspects of food-related pleasure; Table S1: Transcript of questions included in the questionnaire based on The Food Pleasure Scale; Table S2: Overview of distributions and mean values for all variables based on the complete sample, as well as the ‘Moderate stress’ and ‘High stress’ groups. Furthermore, statistical tests and *p*-values are displayed.
