# Supplementary material for: Is Stress Taking the Pleasure Out of Food?—A Characterization of the Food Pleasure Profiles, Appetite, and Eating Behaviors of People with Chronic Stress
Source: Foods. 2022 Jul 4;11(13):1980. doi: 10.3390/foods11131980 (PMC9265269; doi:10.3390/foods11131980)
Supplement: Supplementary file 1 [file foods-11-01980-s001.zip › foods-1788931-supplementary.pdf]

Supplementary material

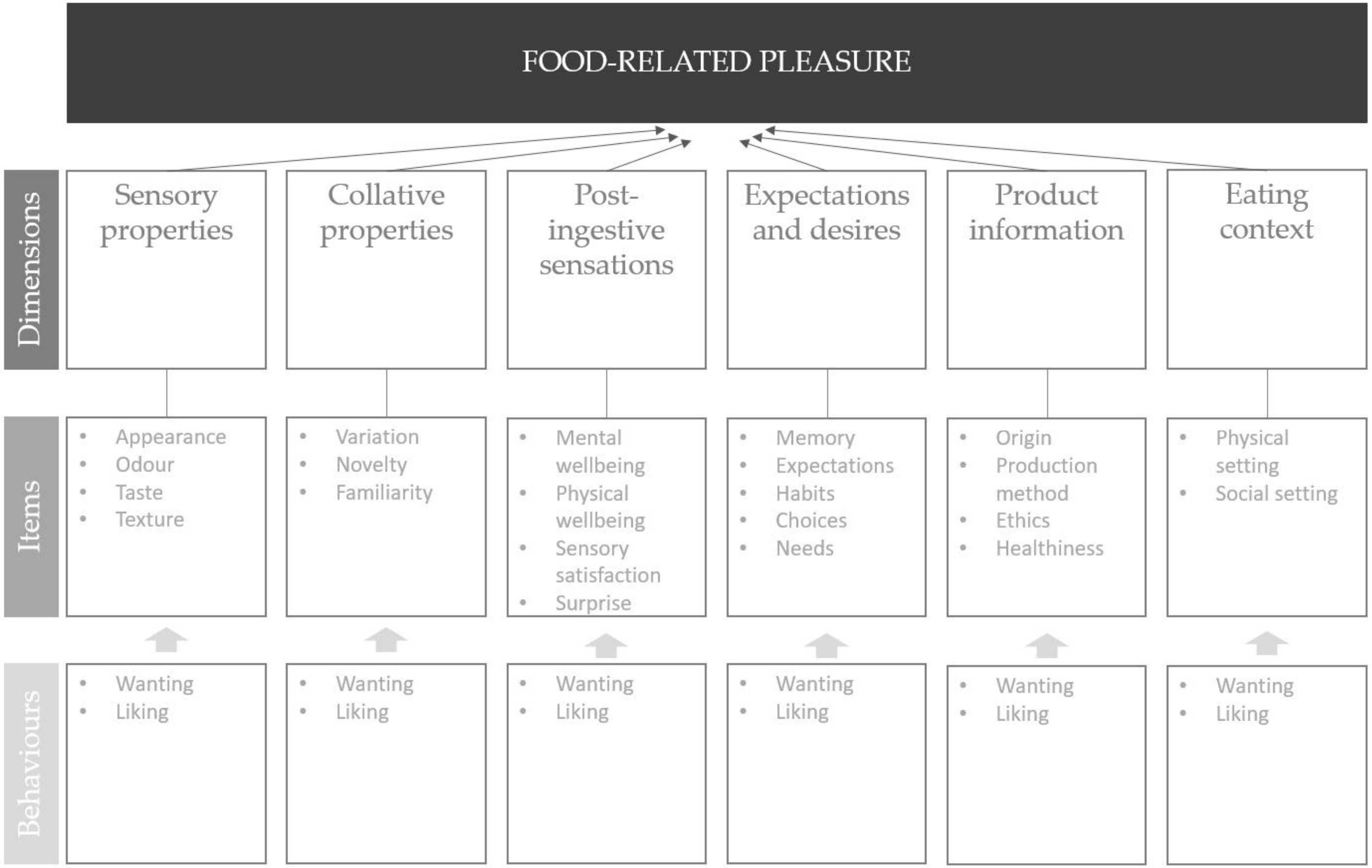

Figure S1. Schematic conceptualisation of the key dimensions, items, and behavioural elements involved in the individual food-related pleasure response, allowing a holistic study of quantitative (level of pleasure) and qualitative (drivers of pleasure) aspects of food-related pleasure. The model is kindly borrowed from Andersen, B. V., Chan, R. C. K., & Byrne, D. V. (2021). A Conceptual Framework for Multi-Dimensional Measurements of Food Related Pleasure—The Food Pleasure Scale. Foods, 10(9), 2044. https://doi.org/10.3390/foods10092044

Table S1. Transcript of questions included in the questionnaire based on The Food Pleasure Scale.

The following questions are about food-related pleasure. First, we want you to assess, which aspects you find pleasure from. Please, try and think of how you feel in the current moment.<br><br>Do you, in this moment, get pleasure from the following aspects of food and meals?		
To experience memories of food?	Yes	No
To have expectations towards the food fulfilled?	Yes	No
To experience the satisfaction of needs?	Yes	No
To experience having choices in the eating situation?	Yes	No
To maintain habits?	Yes	No
To fulfill ethical values about food?	Yes	No
To experience having desired product information about food?	Yes	No
To experience physical surroundings?	Yes	No
To experience food variation?	Yes	No
To experience new/unknown food? (Novelty)	Yes	No
To experience familiar food?	Yes	No
The appearance of the food?	Yes	No
The odor of the food?	Yes	No
The taste of the food?	Yes	No
The texture of the food?	Yes	No
To experience a satisfaction of the sense of sight, smell, taste and touch? (Pleased senses)	Yes	No
To experience eating with others?	Yes	No
To experience eating alone?	Yes	No
To experience physical sensation in the body after eating?	Yes	No
To experience mental sensations after eating?	Yes	No
To experience surprises from the meal?	Yes	No
Now we want you to evaluate how important each aspect is to your general sense of pleasure from food. You do this simply by marking the scale line. Try and think of how you feel in this current moment, and how important each aspect is to you in relation to the other aspects.<br><br>In this moment, how important is the following to your experience of pleasure?		
To experience memories of food?<br><br>Not important	Neither important nor not important	Extremely important
To have expectations towards the food fulfilled?<br><br>Not important	Neither important nor not important	Extremely important

To experience the satisfaction of needs?<br>Not important Neither important nor not important Extremely important
To experience having choices in the eating situation?<br>Not important Neither important nor not important Extremely important
To maintain habits?<br>Not important Neither important nor not important Extremely important
To fulfill ethical values about food?<br>Not important Neither important nor not important Extremely important
To experience having desired product information about food?<br>Not important Neither important nor not important Extremely important
To experience physical surroundings?<br>Not important Neither important nor not important Extremely important
To experience food variation?<br>Not important Neither important nor not important Extremely important

To experience new/unknown food? (Novelty)<br>Not important — Neither important nor not important — Extremely important
To experience familiar food?<br>Not important — Neither important nor not important — Extremely important
The appearance of the food?<br>Not important — Neither important nor not important — Extremely important
The odor of the food?<br>Not important — Neither important nor not important — Extremely important
The taste of the food?<br>Not important — Neither important nor not important — Extremely important
The texture of the food?<br>Not important — Neither important nor not important — Extremely important
To experience a satisfaction of the sense of sight, smell, taste and touch? (Pleased senses)<br>Not important — Neither important nor not important — Extremely important

To experience eating with others?		
Not important	Neither important nor not important	Extremely important
To experience eating alone?		
Not important	Neither important nor not important	Extremely important
To experience physical sensation in the body after eating?		
Not important	Neither important nor not important	Extremely important
To experience mental sensations after eating?		
Not important	Neither important nor not important	Extremely important
To experience surprises from the meal?		
Not important	Neither important nor not important	Extremely important

Table S2. Overview of distributions and mean values for all variables based on the complete sample as well as the 'Moderate stress' and 'High stress' groups. Furthermore, statistical test and p-values are displayed.

	All n=190 (100%)	Moderate stress n=116 (61%)	High stress n=74 (39%)	Test	Statistics	p-value
**Socio-demographic**						
Gender, Female / male				X^2	X^2=1.94, df=1	0.163
Female	155 (82%)	91 (78%)	64 (86%)			
Male	35 (18%)	25 (22%)	10 (14%)			
Age*	44.61 ±11.31	45.43 ±10.79	43.31 ±11.97	Wilcoxon's	W=3879	0.264
Education	5 (3-5)	5 (4-5)	4 (3-5)	X^2	X^2=28.85, df=6	<0.001
- Primary school	5 (3%)	0 (0%)	5 (7%)			
- High School	12 (6%)	4 (3%)	8 (11%)			
- Vocational edu.	43 (22%)	21 (18%)	22 (30%)			
- Short higher edu.	28 (14%)	15 (13%)	13 (18%)			
- Medium higher edu.	68 (36%)	56 (48%)	12 (16%)			
- Long higher edu.	33 (17%)	19 (16%)	14 (19%)			
- PhD	1 (1%)	1 (1%)	0 (0%)			
People in residency	2 (2-4)	3 (2-4)	2 (2-3)	X^2	X^2=9.10, df=8	0.334
**Health and lifestyle**						
PSS-10 score*[2]	25.39 ±4.84	22.29 ±2.96	30.24 ±2.81	Wilcoxon's	W=8584	<0.001
Stress type				X^2	X^2=3.41, df=3	0.333
- Type 1	106 (55%)	67 (58%)	39 (53%)			
- Type 2	22 (12%)	16 (14%)	6 (8%)			
- Type 3	11 (6%)	5 (4%)	6 (8%)			
- Type 4	51 (27%)	28 (24%)	23 (31%)			
Currently on sick leave	126 (66%)	82 (71%)	44 (59%)	X^2	X^2=2.55, df=1	0.112
Confirmed by doctor	159 (84%)	98 (84%)	61 (82%)	X^2	X^2=0.14, df=1	0.709
BMI*[1]	28.00 ±6.40	27.91 ±5.54	28.15 ±7.55	Wilcoxon's	W=3959	0.577
Smoking				X^2	X^2=1.08, df=2	0.583
- Yes	25 (13%)	13 (11%)	12 (16%)			
- No	158 (83%)	99 (85%)	59 (80%)			
- Rarely	7 (4%)	4 (3%)	3 (4%)			
Alcohol consumption				X^2	X^2=1.29, df=2	0.525
- Never	77 (41%)	43 (37%)	34 (46%)			
- 1-2/week	86 (45%)	55 (47%)	31 (42%)			
- 3-4/week	10 (5%)	7 (6%)	3 (4%)			
- 4-5/week	8 (3%)	4 (3%)	4 (5%)			
- 6-7/week	9 (5%)	7 (6%)	2 (3%)			
Extrovert personality	3 (2-4)	3 (2-4)	2 (1.25-4)	X^2	X^2=8.32, df=4	0.081
Introvert personality	3 (2-4)	3 (2-4)	4 (3-4)	X^2	X^2=12.98, df=4	0.011
**Pleasure**						
Snaith-Hamilton Pleasure Scale						

- Normal	98 (52%)	75 (65%)	23 (31%)	X^2	X^2=20.39, df=1	<0.001
- Abnormal	92 (48%)	41 (35%)	51 (69%)	X^2	X^2=20.39, df=1	<0.001
Food Pleasure Scale – I get pleasure from…						
- Memories	73 (38%)	49 (42%)	24 (32%)	X^2	X^2=1.84, df=1	0.175
- Expectations	105 (55%)	69 (59%)	36 (49%)	X^2	X^2=2.15, df=1	0.143
- Needs	112 (59%)	74 (64%)	38 (51%)	X^2	X^2=2.89, df=1	0.089
- Choice	88 (46%)	60 (52%)	28 (38%)	X^2	X^2=3.50, df=1	0.061
- Habits	86 (45%)	57 (49%)	29 (39%)	X^2	X^2=1.80, df=1	0.179
- Ethical values	51 (27%)	32 (28%)	19 (26%)	X^2	X^2=0.08, df=1	0.772
- Product information	43 (23%)	28 (24%)	15 (20%)	X^2	X^2=0.39, df=1	0.534
- Physical surroundings	94 (49%)	59 (51%)	35 (47%)	X^2	X^2=0.23, df=1	0.632
- Variation	101 (53%)	69 (59%)	32 (43%)	X^2	X^2=4.79, df=1	0.029
- Familiarity	142 (75%)	90 (78%)	52 (70%)	X^2	X2=1.28, df=1	0.258
- Novelty	81 (43%)	52 (45%)	29 (39%)	X^2	X^2=0.59, df=1	0.444
- Appearance	129 (68%)	86 (74%)	43 (58%)	X^2	X^2=5.33, df=1	0.021
- Odor	133 (70%)	87 (75%)	46 (62%)	X^2	X^2=3.55, df=1	0.060
- Taste	148 (78%)	95 (92%)	53 (72%)	X^2	X^2=2.77, df=1	0.096
- Texture	103 (54%)	69 (59%)	34 (46%)	X^2	X^2=3.34, df=1	0.068
- Pleased senses	120 (63%)	82 (71%)	38 (51%)	X^2	X^2=7.26, df=1	0.007
- Physical sensation	97 (51%)	67 (58%)	30 (41%)	X^2	X^2=5.36, df=1	0.021
- Mental sensation	78 (41%)	54 (47%)	24 (32%)	X^2	X^2=3.72, df=1	0.054
- Surprise	69 (36%)	46 (40%)	23 (31%)	X^2	X^2=1.44, df=1	0.231
- Eating alone	85 (45%)	54 (47%)	31 (42%)	X^2	X^2=0.40, df=1	0.529
- Eating with others	137 (72%)	90 (78%)	47 (64%)	X^2	X^2=4.45, df=1	0.035
Food Pleasure Scale – importance for pleasure						
- Memories	42.78 ±24.23	44.86 ±23.31	39.51 ±25.25	Wilcoxon's	W=3750.5	0.143
- Expectations	58.40 ±21.98	60.61 ±20.92	54.93 ±23.13	Wilcoxon's	W=3715	0.119
- Needs	59.62 ±21.55	61.37 ±19.79	56.87 ±23.79	Wilcoxon's	W=3734.5	0.132
- Choice	50.42 ±24.60	50.93 ±23.41	49.62 ±26.34	Wilcoxon's	W=4143.5	0.689
- Habits	48.48 ±23.97	48.32 ±24.28	48.73 ±23.47	Wilcoxon's	W=4382.5	0.808
- Ethical values	42.98 ±27.21	45.55 ±26.01	38.95 ±28.53	Wilcoxon's	W=3590	0.058
- Product information	38.70 ±27.65	41.11 ±27.33	34.92 ±27.73	Wilcoxon's	W=3710	0.116
- Physical surroundings	55.09 ±24.74	57.78 ±22.15	50.89 ±27.81	Wilcoxon's	W=3590.5	0.058
- Variation	54.78 ±23.90	58.34 ±23.09	49.21 ±24.09	Wilcoxon's	W=3305.5	0.008
- Familiarity	57.21 ±21.76	57.16 ±21.51	57.28 ±22.14	Wilcoxon's	W=4184	0.771
- Novelty	44.59 ±24.43	45.55 ±22.86	43.10 ±26.64	Wilcoxon's	W=3952	0.358
- Appearance	62.81 ±24.10	65.48 ±20.58	58.64 ±28.26	Wilcoxon's	W=3731.5	0.130
- Odor	64.01 ±22.98	67.91 ±18.73	57.89 ±27.28	Wilcoxon's	W=3247.5	0.0047
- Taste	72.59 ±20.90	75.55 ±18.58	67.94 ±23.35	Wilcoxon's	W=3441	0.021
- Texture	58.61 ±26.36	60.84 ±26.65	55.13 ±25.52	Wilcoxon's	W=3653	0.084
- Pleased senses	64.58 ±22.63	67.56 ±19.51	59.92 ±26.12	Wilcoxon's	W=3510.5	0.035
- Physical sensation	57.96 ±23.63	61.44 ±22.20	52.50 ±24.74	Wilcoxon's	W=3128	0.002
- Mental sensation	57.17 ±25.01	60.53 ±22.57	51.91 ±27.60	Wilcoxon's	W=3395	0.015
- Surprise	42.02 ±24.16	43.26 ±23.78	40.09 ±24.63	Wilcoxon's	W=4036.5	0.490

- Eating alone	45.05 ±24.70	42.40 ±22.63	49.20 ±27.12	Wilcoxon's	W=4943.5	0.078
- Eating with others	57.54 ±26.45	57.95 ±24.23	56.90 ±29.58	Wilcoxon's	W=4284.5	0.985
**Food preference changes**						
Fruit and vegetables				X^2	X^2=8.57, df=2	0.014
- Less	70 (37%)	34 (29%)	36 (49%)			
- Same	95 (50%)	63 (54%)	32 (43%)			
- More	23 (12%)	18 (16%)	5 (7%)			
Meat and meat products				X^2	X^2=1.49, df=2	0.474
- Less	31 (16%)	16 (14%)	15 (20%)			
- Same	150 (79%)	95 (82%)	55 (74%)			
- More	8 (4%)	5 (4%)	3 /4%)			
Milk and dairy products				X^2	X^2=0.49, df=2	0.783
- Less	27 (14%)	18 (16%)	9 (12%)			
- Same	152 (80%)	92 (79%)	60 (81%)			
- More	9 (5%)	5 (4%)	4 (5%)			
Bread, pasta, rice, potatoes				X^2	X^2=2.28, df=2	0.320
- Less	21 (11%)	11 (9%)	10 (14%)			
- Same	167 (72%)	89 (77%)	48 (65%)			
- More	30 (16%)	16 (14%)	14 (19%)			
Salty snacks				X^2	X^2=0.73, df=2	0.693
- Less	33 (17%)	20 (17%)	13 (18%)			
- Same	96 (51%)	61 (53%)	35 (47%)			
- More	60 (32%)	34 (29%)	26 (35%)			
Sweet treats				X^2	X^2=2.41, df=2	0.300
- Less	24 (13%)	18 (16%)	6 (8%)			
- Same	68 (36%)	39 (34%)	29 (39%)			
- More	97 (51%)	58 (50%)	39 (53%)			
Fast-food and take-away				X^2	X^2=0.87, df=2	0.648
- Less	26 (14%)	18 (16%)	8 (11%)			
- Same	85 (45%)	52 (45%)	33 (45%)			
- More	78 (41%)	46 (40%)	32 (43%)			
Home-cooked meals				X^2	X^2=5.52, df=2	0.063
- Less	64 (34%)	32 (28%)	32 (43%)			
- Same	105 (55%)	68 (59%)	37 (50%)			
- More	20 (11%)	15 (13%)	5 (7%)			
Soft drinks				X^2	X^2=4.42, df=2	0.110
- Less	27 (14%)	21 (18%)	6 (8%)			
- Same	116 (61%)	70 (60%)	46 (62%)			
- More	47 (25%)	25 (22%)	22 (30%)			
Coffee and Tea				X^2	X^2=8.01, df=2	0.018
- Less	40 (21%)	26 (22%)	14 (19%)			
- Same	115 (61%)	76 (66%)	39 (53%)			
- More	35 (18%)	14 (12%)	21 (28%)			
Alcohol				X^2	X^2=1.29, df=2	0.525

- Less	69 (36%)	39 (34%)	30 (41%)			
- Same	97 (51%)	63 (54%)	34 (46%)			
- More	24 (13%)	14 (12%)	10 (14%)			
Main meals				X^2	X^2=10.37, df=2	0.006
- Less	58 (31%)	26 (22%)	32 (43%)			
- Same	115 (61%)	76 (66%)	39 (53%)			
- More	16 (8%)	13 (11%)	3 (4%)			
Snack meals				X^2	X^2=8.93, df=2	0.012
- Less	63 (33%)	30 (26%)	33 (45%)			
- Same	73 (38%)	53 (46%)	20 (27%)			
- More	53 (28%)	33 (28%)	20 (27%)			
**Appetite**						
General desire for food	3 (2-4)	3 (2-4)	2 (2-4)	X^2	X^2=12.03, df=4	0.017
Meals before stress						
- Breakfast	159 (84%)	101 (87%)	58 (78%)	X^2	X^2=2.41, df=1	0.114
- Pre Lunch Snack	67 (35%)	43 (37%)	24 (32%)	X^2	X^2=0.43, df=1	0.514
- Lunch	182 (96%)	112 (97%)	70 (95%)	X^2	X^2=0.43, df=1	0.512
- Pre Dinner Snack	107 (56%)	69 (59%)	38 (51%)	X^2	X^2=1.21, df=1	0.271
- Dinner	188 (99%)	115 (99%)	73 (99%)	X^2	X^2=0.10, df=1	0.747
- Post Dinner Snack	87 (46%)	47 (41%)	40 (54%)	X^2	X^2=3.34, df=1	0.068
- Night meal	3 (2%)	1 (1%)	2 (3%)	X^2	X^2=0.98, df=1	0.321
Meals now						
- Breakfast	132 (69%)	87 (75%)	45 (61%)	X^2	X^2=4.29, df=1	0.038
- Pre Lunch Snack	73 (38%)	47 (41%)	26 (35%)	X^2	X^2=0.55, df=1	0.457
- Lunch	140 (74%)	90 (78%)	50 (68%)	X^2	X^2=2.34, df=1	0.126
- Pre Dinner Snack	111 (58%)	72 (62%)	39 (53%)	X^2	X^2=1.63, df=1	0.202
- Dinner	184 (97%)	116 (100%)	68 (92%)	X^2	X^2=9.71, df=1	0.002
- Post Dinner Snack	102 (54%)	59 (51%)	43 (58%)	X^2	X^2=0.95, df=1	0.329
- Night meal	16 (8%)	8 (7%)	8 (11%)	X^2	X^2=0.90, df=1	0.343

*Mean ± standard deviation, [1]BMI: Body Mass Index, [2]PSS-10: Perceived Stress Scale. Moderate stress level corresponds to a score of 14-26, High stress level to a score of 27-40.
